# Searching for BRCA3 by exome sequencing

**DOI:** 10.1186/1897-4287-10-S2-A68

**Published:** 2012-04-12

**Authors:** I Makunin, M Stark, M Gartside, G Chenevix-Trench

**Affiliations:** 1 Queensland Medical Research Institute, Brisbane and Peter MacCallum Cancer Centre, Melbourne, Australia

## 

The current paradigm suggests that some non-*BRCA1/2* multiple case breast cancer families are caused by rare mutations in high-risk genes. We are using exome sequencing to identify putative ‘BRCA3’ genes in a small number of kConFab families. We selected five non-*BRCA1*/2 families containing 5-9 breast cancer cases (of which 0-4 per family were affected under the age of 40) who have had extensive analysis of *BRCA1* and *BRCA2*, and for which germline DNA was available from two distant relatives (at least 4^th^ degree) affected with breast cancer between 30 and 56 years of age. In first stage we isolated the exomes of two pairs of affected individuals (4^th^ and 5^th^ degree relatives, from two families) diagnosed with breast cancer between the age of 30 and 39, using the SeqCap EZ exome capture kit (NimbleGen) and then sequenced the libraries on the GAIIx and HiSeq (Illumina) platforms with a paired-end protocol. The sequences were aligned to the human genome with BWA and we then compared several pipelines for processing the sequences and calling variants. The best results were obtained with the Picard/GATK pipeline after local re-alignment and re-calibration of the base quality. The samples from the remaining three families will be sequenced by Axeq Inc. and the results of the analysis will be presented.

